# The value of thyroperoxidase as a prognostic factor for differentiated thyroid cancer -- a long-term follow-up study

**DOI:** 10.1186/s13044-015-0022-6

**Published:** 2015-08-13

**Authors:** Yurena Caballero, Eudaldo M. López-Tomassetti, Julián Favre, José R. Santana, Juan J. Cabrera, Juan R. Hernández

**Affiliations:** General Surgery Department, Hospital Universitario Insular de Gran Canaria, Las Palmas de Gran Canaria, Las Palmas, Spain; Pathology Department, Hospital Universitario Insular de Gran Canaria, Las Palmas de Gran Canaria, Las Palmas, Spain

**Keywords:** Thyroid cancer, Thyroperoxidase, Ki67, p53

## Abstract

**Background:**

Thyroperoxidase (TPO) is a membrane-bound protein essential for the production of thyroid hormones; because of this, TPO expression may be impaired in selected thyroid diseases. The goal of this study is to analyze TPO immune expression in differentiated thyroid cancer, and to determine whether TPO has any prognostic value.

**Methods:**

A total of 139 patients who required surgery due to a thyroid nodule with signs or symptoms suspicious for malignancy during their physical, ultrasound and/or cytology examination were consecutively selected for the study. A study of TPO immunohistochemical expression was carried out on these patients using the MoAb47 monoclonal antibody. In addition, cell proliferation marker *Ki67* and tumor suppressor *p53* were also measured for comparison.

**Results:**

A total of 139 cases, 43 benign tumors, 42 papillary carcinomas, 38 follicular carcinomas, 8 undifferentiated carcinomas, and 8 sporadic medullary carcinomas were analyzed. The relationship between TPO expression and disease was statistically significant (*p* <0.001), and decreased with tumor dedifferentiation extent. Increased TPO expression in benign lesions as compared to decreased expression in papillary carcinomas and undifferentiated tumors is outstanding. Differences in TPO expression were observed in minimally invasive follicular carcinoma (MIFC) compared to widely invasive follicular carcinoma (WIFC). TPO expression decreases in undifferentiated malignancies in contrast with *p53* and *Ki67* expression, which increases in that setting. TPO, *p53* and *Ki67* expression was significantly related to TNM stage (*p* <0.001). Survival rate was 72 % after a 20-year follow-up, and 100 % for subjects with higher TPO expression.

**Conclusions:**

TPO may be useful in confirming or ruling out benign diseases from differentiated thyroid carcinoma, with the exception of low-risk carcinoma such as MIFC. It could be used as a prognostic factor for differentiated thyroid cancer and patient follow-up, together with other markers.

## Background

Thyroperoxidase (TPO) is a membrane-bound protein essential for thyroid hormone production, characteristic of functional, normal thyroid cells. TPO expression is considered a thyroid differentiation marker. Qualitative and quantitative alterations in TPO activity, TPO messenger ribonucleic acid (mRNA), and protein expression can be related to thyroid changes and have been reported in pathological thyroid tissues [1]. TPO content has been shown to be significantly lower in thyroid malignancies as compared to benign conditions and normal tissue [2]; also, a reduction in TPO activity and TPO mRNA of 55–70 % is observed in differentiated thyroid carcinoma (DTC) [1, 3]. Moreover, anaplastic tumors have non-existent TPO expression. The biological meaning of this abnormal TPO expression is unclear; however, a deregulation of protein synthesis can be related. A progressive decrease in TPO levels, together with increased cell density, suggests an association with proliferation.

Molecular tests are emerging that measure different gene and protein combinations that may differentiate benign lesions from DTC [4–6]. Although these studies have shown good results and can be promising for the future, these techniques are not readily available in every hospital. On the other hand, immunohistochemistry techniques could be an easier option to differentiate between benign thyroid lesions and DTC.

Many studies have described immunohistochemical markers that improve the diagnosis of thyroid conditions such as TPO on their own or, alternatively, associated with other markers such as *galectine-3, CK19, HBME-1,* etc. De Micco determined that anti-TPO antibody MoAb47 recognized TPO expression in normal and benign thyroid tissues, but only in 3 % of malignant tumors [7]. TPO gene and protein expression in thyroid carcinoma have been analyzed, indicating low enzymatic activity [2], impaired solubility and suppressed TPO mRNA expression [8]. Savin et al. studied the value of TPO combined with *galectine-3* in DTC, observing that TPO had an intense expression in normal or hyperplasic thyroid tissue, and was down-regulated in thyroid pathologies. They reported an inverse correlation with known prognostic factors and TNM staging [9]. However, other reports have obtained the same TPO expression in both benign thyroid diseases and DTC [10].

This study analyzes the immunohistochemical expression of TPO (using MoAb-47) in both benign and malignant lesions to establish the relationship between TPO expression, histological type, differentiation degree, and tumor growth. In malignancies, including both differentiated and undifferentiated cancers; a comparative analysis of TPO with proliferation factor *Ki67* and cell-cycle suppressor protein *p53* was carried out [11–13].

## Methods

Patients with thyroid nodules and signs or symptoms suspicious for malignancy during physical, ultrasound and/or cytology examination who required surgery during the period 1972 to 1995 were consecutively selected for this study. The ethical approval and informed consent of patients were obtained (The institutional review board was approved by the committe of the Hospital Universitario Insular de Gran Canaria and the University of Las Palmas of Gran Canaria). The institutional review board was approved by the committe of the Hospital Universitario Insular de Gran Canaria and the University of Las Palmas of Gran Canaria. Only 139 cases were included as we had a limited number of monoclonal antibodies available for the TPO immunohistochemical study. Data was prospectively collected and patients were divided into four groups according to their histological diagnosis: benign cases, papillary thyroid carcinoma (PTC), follicular thyroid carcinoma (FTC), and a fourth group called “others”, which included undifferentiated thyroid carcinomas and sporadic medullary carcinomas. Specific variants were included in the study, such as the follicular variant of papillary carcinoma and Hürthle cell carcinoma for the PTC group, clear cells and insular pattern for the FTC group. Even though sporadic medullary carcinomas are related to C-cells (and therefore to calcitonin levels) they were not excluded, as we wanted to evaluate TPO, *p53* and *ki67* expression.

### Histological analysis

After gross examination, the specimens were fixed in 10 % formaldehyde and embedded in paraffin. Blocks were cut using a Leica microtome into 4–5 micron sections, and then studied with several staining techniques (Harris or Mayer hematoxylin, eosin, PAS).

### Immunohistochemistry

The antibodies used for the immunohistochemical study included MoAb-47 for TPO, DO-7 for *p53*, and *Ki67* -MM1 against antigen *Ki67*.

Once obtained, histology blocks were placed in the heater for twelve hours at 37 °C and then soaked in xilol three times over 15 min, twice in absolute alcohol over 10 min, once over 5 min in 96 % alcohol, once in 70 % alcohol, and lastly once time during 5 min in distilled water. Afterwards, all slides were placed in a pressure cooker with citrate buffer solution as a recovery antigenic system. A process to inhibit endogenous peroxidase using 250 ml of 30 % hydrogen peroxide with 10 ml of methanol during 10 min followed this. Antibody solutions were prepared with the following dilutions: TPO 1:50, p53 1:50, and ki67 1:100; these were maintained at a temperature of 4 °C during the staining process. Then we started the incubation process without washing the slides with the primary antibody during 1 h, with the secondary antibody during another hour, and with the avidin-biotin-peroxidase complex during 30 min.

In the final stage, slides were washed in PBS for 15 min with agitation, and subsequently developed with chromogenic substrate for 3 to 6 min away from direct light (diaminobenzidine [DAB] solution); then they were washed with tap water for 5 min 3 times, and contrasted with Harris’ or Mayer’s hematoxylin for 1 min; finally they were dehydrated using a growing alcohol series (70 %, 96 %, and 100 % twice, and xylol clearing solution twice), and eventually mounted with coverslips using DPX).

Routine techniques such as Congo red under polarized light and calcitonin immunohistochemistry were used for sporadic medullary carcinomas.

### Immunohistochemistry assessment

TPO expression was measured under a light microscope, in terms of percentage of stained cells and stain intensity, using different scores. For the percentage of stained cells an Arabic number was given: 0, 1–10 % stained cells = 1, 10–33 % = 2, 33–66 % = 3, >66 % = 4; and for stain intensity: negative = 0, mild = 1, moderate = 2, intense = 3. Intensity and proportion of stained cells were summed using the Vargas-Roig method [14] in order to obtain a single result. Thus, a tumor with a total result of 0–2 was classified as negative, one with 3–4 as mild positive, one with 4–5 moderately positive, and one with 5–7 as intensely positive. One pathologist, who was blinded to cytology results, was in charge of the immunohistochemical expression measurement. Separately, another pathologist studied the surgical specimen and provided a final histological diagnosis. The scoring was performed with blinding to patient outcome.

Furthermore, mean, standard deviation, and 95 % confidence interval values were found for each area, both regarding percentage and intensity. The morphological aspects of immunostains were also described for the different pathologies. Overall survival was analyzed according to TPO level and TNM stage, as well as disease-free survival. Disease-free survival was considered for DTC when there was no clinical evidence of tumor, no imaging evidence of tumor, and undetectable serum thyroglobulin levels during TSH suppression and stimulation in the absence of interfering antibodies. For medullary thyroid carcinoma, calcitonin serum levels and carcinoembryonic antigen (CEA) levels were tested every 6 months during the first year, and then annually with an imaging procedure for control.

### Statistical analysis

Data was analyzed using the SPSS v. 18.0 software. Statistical techniques included Pearson’s squared chi test for the study of association between categorical variables; Student’s *t*-test in the comparison of means for normally distributed numerical variables; Wilcoxon’s non-parametric test in case of non-normality; a binary logistic regression model to find parameter profiles and TPO, *Ki67*, and *p53* values; and a Cox proportional hazard analysis for assessing the effect of each prognostic factor on overall survival. A significance level of 5 % (*p* <0.05) was established for all tests.

## Results

This series includes a total of 139 cases, 43 benign and 96 malignant, classified in four groups. Table 1 summarizes the histological diagnoses by group. The surgical procedures performed included lobectomies, subtotal thyroidectomies (this type of procedure was carried out in patients from 1972 to 1995), and total thyroidectomies.

**Table 1:** Diagnosis by group

Group		Number (N)
Benign	Nodular hyperplasia	
	- Multinodular	10
	- Uninodular	9
	Chronic thyroiditis	
	- Hashimoto	7
	- Chronic nonspecific lymphocytic thyroiditis	3
	Adenoma	16
	Total	43
Papillary carcinoma	Papillary Carcinoma	38
	Follicular variant	4
	Total	42
Follicular carcinoma	Widely invasive	28
	- Clear cells	2
	- Hürthle cells	2
	- Insular pattern	5
	Minimally invasive	10
	Total	38
Other	Undifferentiated carcinoma	8
	Sporadic medullary carcinoma	8
	Total	16

**Fig. 1:**
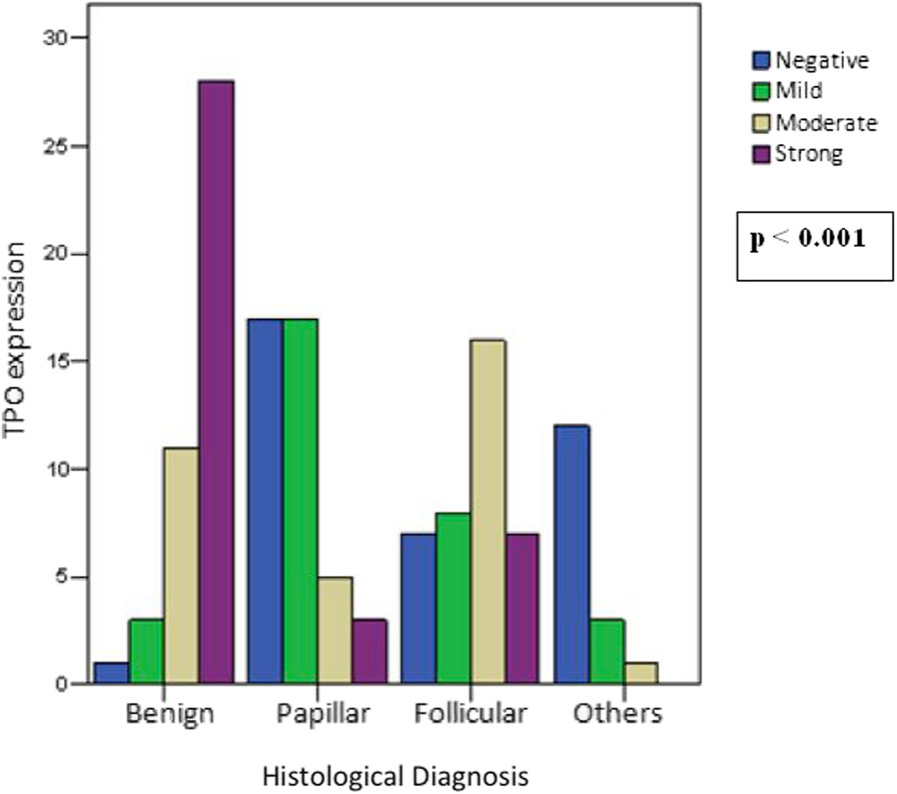
TPO immunohistochemical expression according to tumor histology. TPO by groups showed an intense expression of TPO in benign cases when compared to the rest of groups, decreasing significantly in the papillary group, as well as in undifferentiated and sporadic medullary tumors (group “others”), where it was mainly negative. In the follicular group, TPO expression was variable, showing positive TPO expression but not as much as in the benign cases

**Fig. 2:**
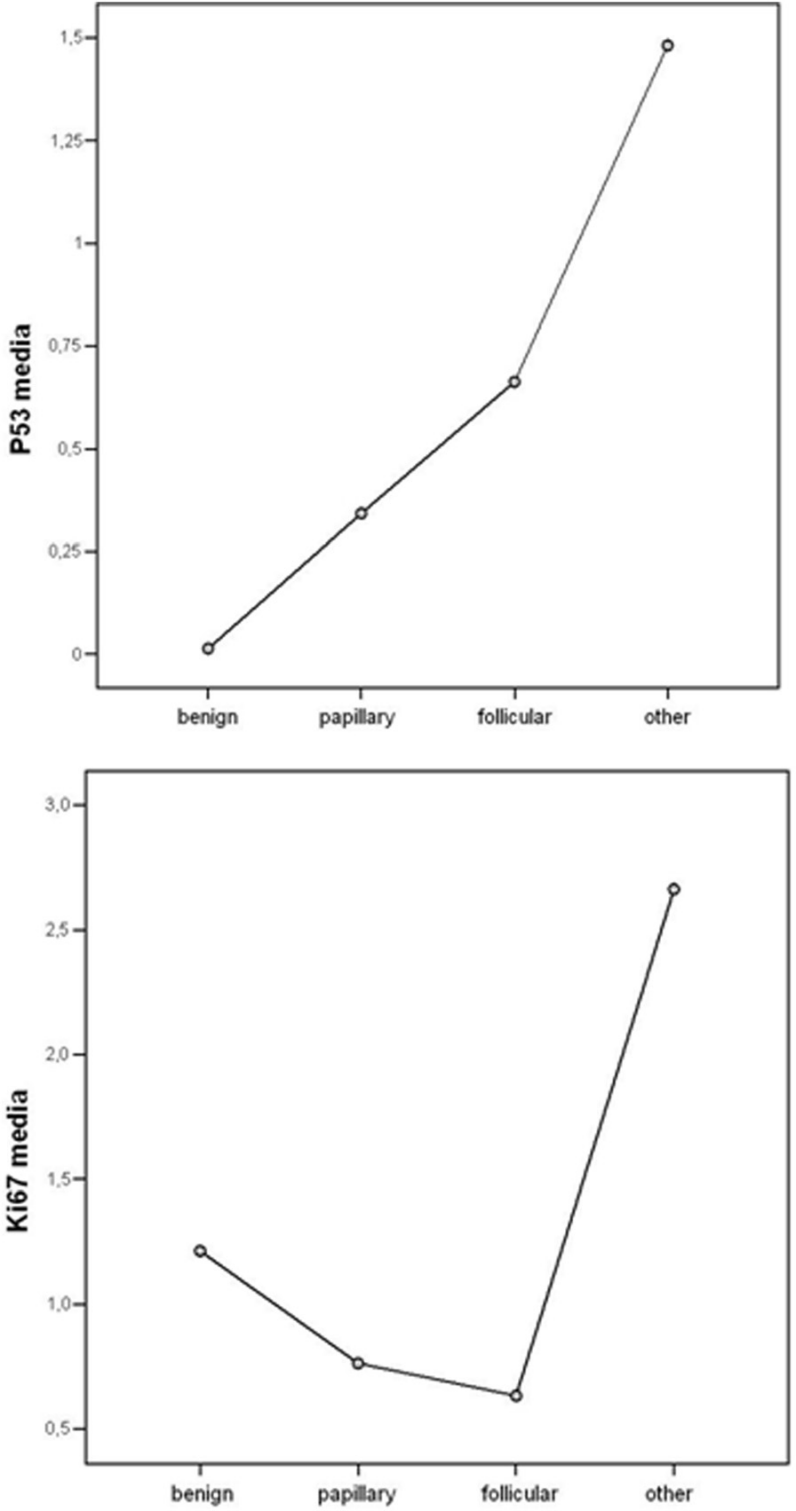
p53 and ki67 levels by histological group. The figure at the *top* shows p53 expression by group, with benign cases showing a negative expression. Papillary and follicular carcinomas had a mild to moderate positive expression, with increasing intensity in the undifferentiated cases. In the figure at the *bottom*, ki67 showed an intermediate positive expression in benign cases, with an equal proportion of mild, moderate and negative cases in papillary carcinomas, then dropping to scantly positive in follicular cases and arising to intense positive in undifferentiated carcinomas

The immunohistochemical expression of TPO, *p53* and *Ki67* by disease is detailed in Figs. 1 and 2. TPO expression related to histological diagnosis was statistically significant (*p* <0.001), with expression decreasing with cellular dedifferentiation. TPO staining patterns were also found in the different groups.

### Benign pathology

**Fig. 3:**
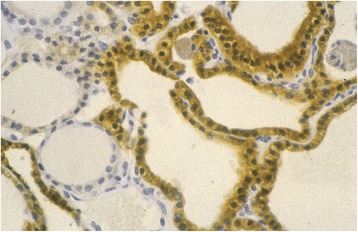
Multinodular hyperplasia: Nodular hyperplasia showing cytoplasmic TPO expression (TPO x20) -- highly intense in small follicles and apical areas

In multinodular hyperplasia TPO was consistently positive in the cytoplasm (TPO staining scores: 2.7–5.5), with an irregular distribution highly intense in small follicles with apical TPO concentrations (Fig. 3). Uninodular cases showed moderate to high immunostaining (3.9 to 6.0) in the cytoplasm, particularly in microfollicular areas with cytoplasmic granules, as may be expected from a benign disease. A direct association between TPO intensity, presence of microfollicles, and localization in areas near the capsule was found. In contrast, *p53* and *Ki67* expression was mostly negative.

TPO expression was moderate to high in all adenomas (mean 4.5), regularly distributed in the cytoplasm with a marked apical predominance. In Hürthle-cell adenomas low TPO positivity is characteristic both in central and subcapsular follicles; positivity is only outstanding for papillary growth patterns. The histological study of *p53* was negative in all but two cases -- microfollicular and embryonic types. *Ki67* expression had intermediate positivity (1.2 to 2.4) in normal follicular, microfollicular and Hürthle-cell adenomas, with higher levels in a trabecular adenoma.

### Papillary thyroid carcinoma

**Fig. 4:**
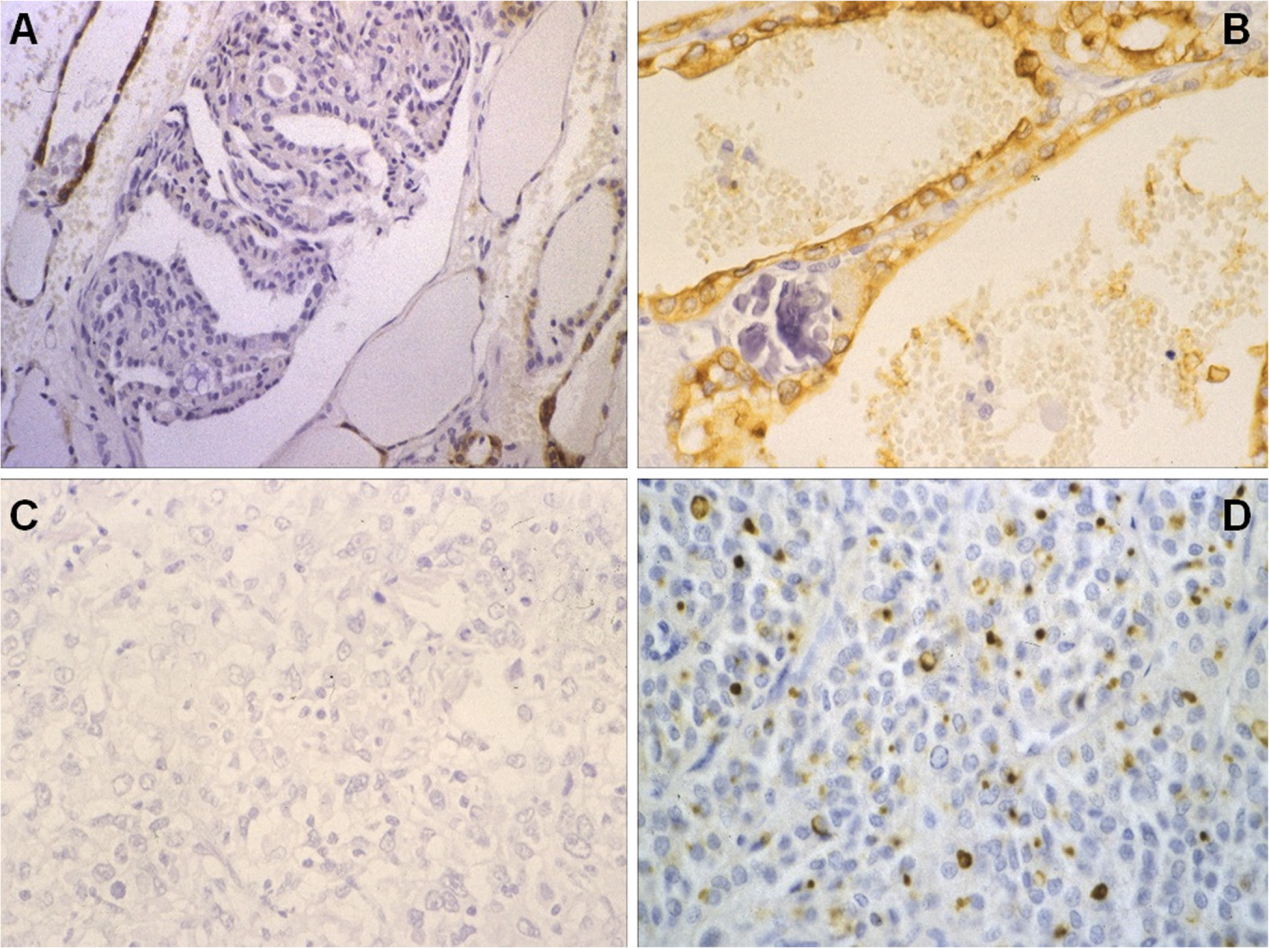
**a** Well-differentiated follicular carcinoma: TPO expression appears positive in normal thyroid cells and negative in tumor cells (TPO x20); **b** Papillary carcinoma: positive TPO expression in the apical portion of epithelial cells and negative in Psammoma bodies (TPO x40); **c** Undifferentiated carcinoma: negative TPO expression (TPO x40); **d** Medullary carcinoma: expression of TPO in cytoplasmic vesicles (TPO x20)

TPO staining was usually poor to moderate (<3) in 81 % of cases -- higher TPO scores were only seen in four patients (3.6 and 6) -- with positive expression in the apical pole of cells in the cystic epithelium and negative expression in Psammoma bodies (Fig. 4b). In follicular variants, negativity was seen in most fields except for focal areas with a regular and patchy distribution.

Protein *p53* had mild to moderate staining overall, and *Ki67* showed an equal distribution of mild, moderate and negative staining.

### Follicular thyroid carcinoma

TPO results revealed positivity with irregular expression in most cases. Minimally invasive follicular cases (MIFC) (3.5 and 4.9) showed greater TPO immunostaining as compared to widely invasive follicular cases (WIFC) (1.4 and 2.9), with the presence of extensive, totally negative areas in contrast to their neighboring lesion zones (Fig. 4a). Five cases were totally negative. FTC showed decreased TPO immunostaining, both in intensity and percentage, when compared to hyperplasia and, to a lesser extent, thyroid adenoma. These lesions had an irregular and cytoplasmic TPO stain, with mild and intense areas. In MIFCs TPO expression was moderate to intense in the cytoplasm and mild or negative in the nuclei; intracellular distribution was uniform from basal to apical areas. The presence of TPO in infiltrating follicles, including those protruding into the vascular lumen, is interesting. In WIFCs, expression was usually poorer.

*P53* was negative for most MIFCs and positive in three Hürthle-cell carcinomas (1.5–3); WIFCs had scant positivity, and both clear-cell and insular-pattern lesions were negative. *Ki67* was scarcely positive in both MIFCs and WIFCs (0.4 and 1.3), with insular variants being moderately positive (1.4 and 3.7).

### Others

#### Non-differentiated carcinoma

TPO was negative except in one case, which was considered follicular reclassified as anaplastic. *P53* positivity was generally intense (4 and 6.5), and *Ki67* was positive in all cases with variable intensity (0.4 and 5.8), with anaplastic variants being more intensely positive (Fig. 4c).

#### Sporadic medullary carcinoma

TPO was negative in most cases and moderate in three; the presence of TPO in cytoplasmic vesicle-like deposits was noteworthy (Fig. 4d). *P53* immunostaining was negative except for two cases where it was markedly weak (0.2), and *Ki67* was negative in four cases and mild to moderate in the remaining four (2 y 4).

**Fig. 5:**
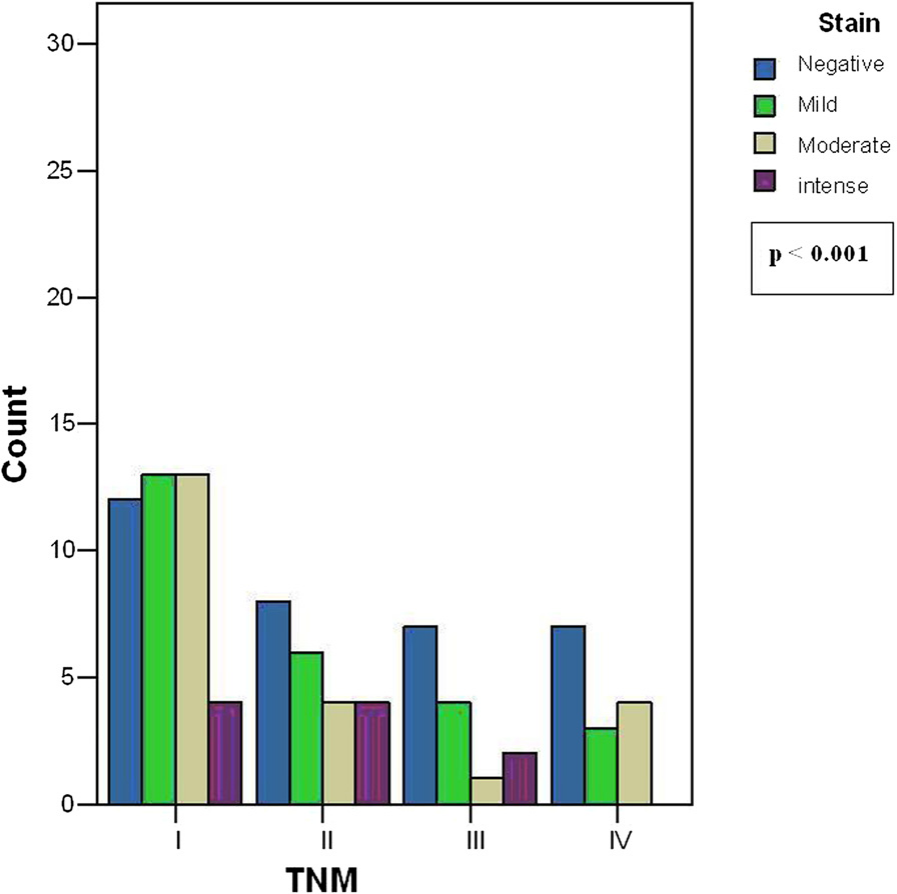
Relationship between TPO level and TNM stage: The figure shows how TPO expression decreases when TNM increases in terms of intensity, as in stage I more positive than negative cases are found, and in stage II, III and IV more negative than positive cases are observed

The cancer cohort was classified according to TNM stage. Figure 5 illustrates the association between TPO immunostaining and TNM stage, observing that TPO decreases as TNM increases.

**Fig. 6:**
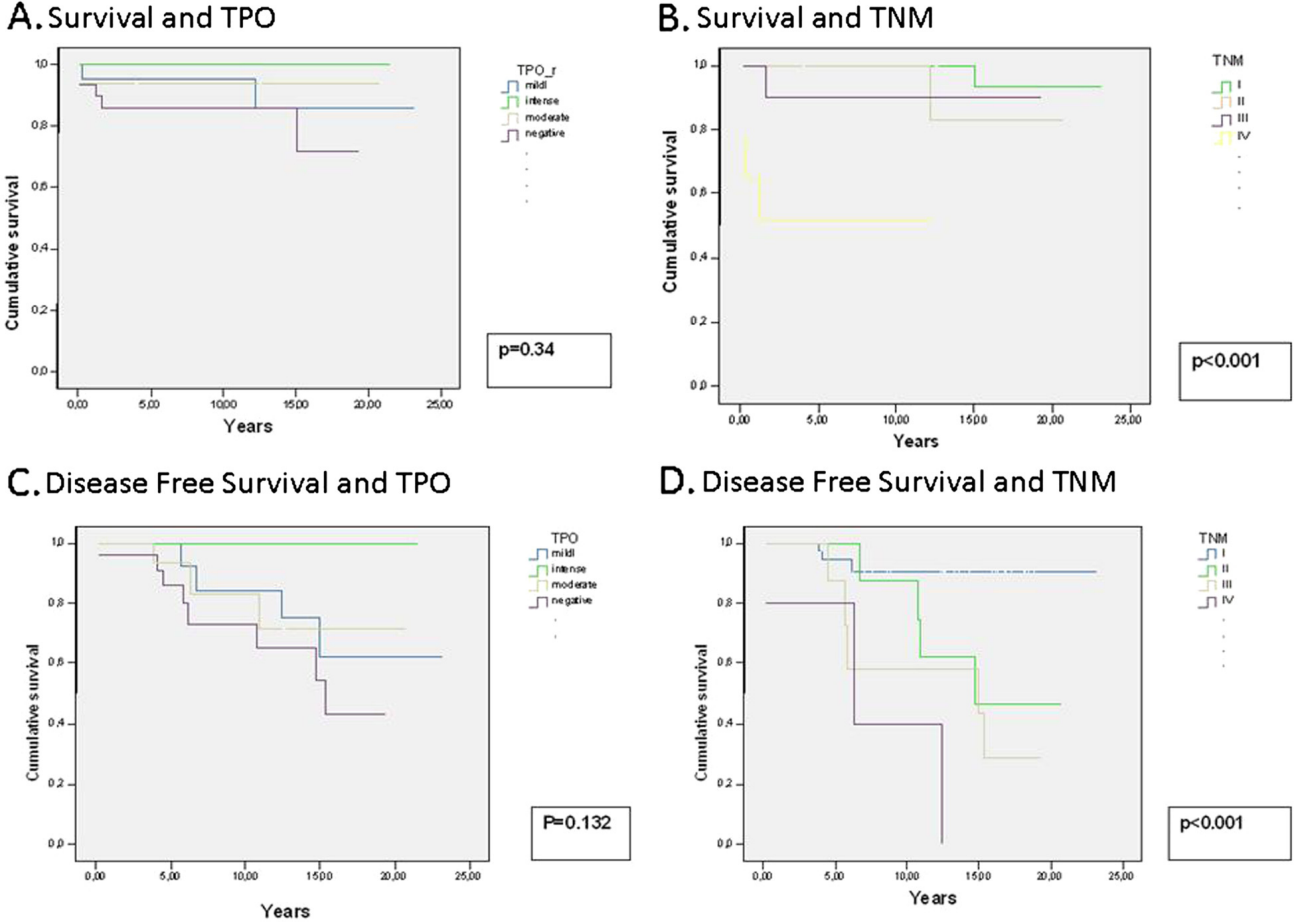
**a** Overall survival according to TPO level: overall survival decreases with TPO expression. **b** Overall survival according to TNM stage: Overall survival decreased with TNM stage, with a 20-year survival of 90 %, 83 % and 52 % in stages II, III and IV, respectively. **c** Disease-free survival according to TPO expression: **d** Disease-free survival according to TNM stage: The figure shows how disease-free survival (DFS) decreased with TNM stages, with 95 % in stage I, 47 % in stage II, 29 % in stage III, and 0 % in stage IV

Follow-up was carried out on an outpatient basis by the endocrinologist and the surgeon by way of physical examination, yearly thyroglobulin levels, and ultrasounds for suspected relapse. A maximum follow-up of 20 years was carried out. Minimum and median follow-up was 5 and 15 years, respectively. A survival study was carried out, limited to patients with cancer, according to total TPO level (Fig. 6a) and TNM stage (Fig. 6b). Disease-free survival related to TPO expression (Fig. 6c) and TNM stage (Fig. 6d) was also analyzed.

According to TPO level, overall survival decreased in negative cases compared to positive cases without reaching statistical significance (*p* = 0.34). On the other hand, survival decreased with higher TNM stages (*p* <0.001), as patients in stage IV had a 20-year survival of 52 % (SD 0.18) compared to patients in stage III and II, who had a 20-year survival of 83 % (SD 0.15) and 90 % (SD 0.09), respectively. We did not find any reason for the different survival rates in stages II and III, as they are differentiated by patient age and tumor size and localization.

Disease-free survival also decreased significantly in cases with higher TNM stages (*p* <0.001). Patients in stage I had a 95 % disease-free survival after a 20-year follow-up. This rate dropped to 47 % for stage II, where patients under the age of 45 are included without taking into account tumor size or local or distant invasion. Stage III had a 29 % disease-free survival, whereas for stage IV all patients were ill at the end the observation period.

## Discussion

TPO is a membrane-bound protein essential in the production of thyroid hormones. It catalyzes the iodination and coupling of tyrosyl residues to thyroglobulin to form thyroid hormones T3 and T4. TPO expression pattern correlates with thyroid follicular cell function, both at the cytoplasm and apical membrane compartments. Quantitative and qualitative changes are related to hormone biosynthesis abnormalities usually due to thyroid disease. The biological meaning of abnormal TPO expression in thyroid tumors is unclear, but the progressive decrease of TPO levels together with an increase in cell density suggests it is correlated with proliferation [1–3].

*Ki67* was used to study the proliferative activity of tumors, which determines their aggressiveness, progression, and metastatic potential. It is a monoclonal antibody that binds an antigen present in proliferating cells, but absent from quiescent cells [11]. On the other hand, gene *p53* is a suppressor gene that codes for a *p53* protein that plays a role in cell-cycle control, replication, and DNA repair. Mutated *p53* accumulates in the nucleus of tumor cells, and is promptly identifiable using immunohistochemical techniques [12, 13].

A huge variety of molecular tests are now available for the measurement of prognostic factors in thyroid carcinoma. Weber [4] identified a 3-gene combination to differentiate follicular adenomas from follicular carcinomas, with 100 % sensitivity and 97 % specificity. Rosen [5] compared gene expression in benign thyroid lesions versus papillary carcinoma, and described a set of 6 genes that predicted pathological diagnosis with 75 % sensitivity and 100 % specificity; Kroll [6] described fusion protein *PAX8/PPAR-γ* as a marker to distinguish benign from malignant follicular lesions. However, molecular techniques and gene studies are costly and time-consuming procedures that render their routine use difficult.

Our study’s approach is different. Using simpler methods, such as immunohistochemistry, we analyzed TPO, *p53* and *Ki67* expression with the purpose of assessing whether TPO is useful as a prognostic marker by means of a long-term survival study. We have not found any literature describing a single study with such a long follow-up. The relationship between TPO expression and disease was statistically significant (*p* <0.001) and decreased with tumor dedifferentiation extent. We do not know exactly why TPO is lost or increased according to tumor growth or development, albeit we suspect that differentiation criteria are involved. Increased TPO expression in benign lesions as compared to PTC and undifferentiated tumors is outstanding.

TPO increased in FTC to a lesser extent than in benign lesions, with differences in TPO expression between MIFC and WIFC being consistent with most reported studies [7, 15–17]. However, others such as Savin et al. among 47 cases of FTC observed an overall TPO expression of 78.7 % without correlation with degree of histopathological aggressiveness, as TPO expression in WIFC was not less reactive than in MIFC [9]. Microfollicular adenomas and MIFCs show increased TPO levels, which may reflect a failure of these invasive cells to lose function and differentiation despite their neoplastic course.

In contrast, De Micco [7, 15] observed that immunostaining was irregular in adenomas, moderate in 40 % of the remaining diseases, and negative in follicular carcinomas. This report does not include morphological criteria, immunostaining differences across areas, cytoplasmic localization, intensity assessment, or percentage per histological fields. Weber [4], in a series of 9 FTCs, found that only one was negative, thus reporting high positivity levels and a low sensitivity of TPO for this type of malignancy. In another study, Weber analyzed the use of TPO and galectine-3 separately for the diagnosis and prognosis of thyroid cancer — also combining both markers — and obtained with TPO staining a sensitivity of 39 % for any cancer, of 50 % for PTC, and of 11 % for FTC, which increased with galectine-3 immunostaining to 82 %, 96 % and 44 %, respectively [18]. Therefore, follicular cancer is associated with abnormal TPO expression in studies, with a decrease in TPO immunostaining in 80–95 % of follicular carcinomas.

The scarce TPO staining of PTC in our series is consistent with other reports [15, 19, 20] that show strong TPO suppression in papillary carcinomas. The negative TPO expression found in undifferentiated carcinomas, where cells lack their endocrine function, supports an association between cell differentiation and TPO levels.

BRAF^V600E^ mutation is one of the most common genetic alterations in thyroid carcinogenesis. Its presence in papillary carcinomas has been reported to be associated with advanced stages [21] and poorer clinical outcome, although this remains controversial. Romei et al. analyzed the mRNA expression levels of TPO, among others, according to the presence of BRAF^V600E^ mutation [22]. They observed a higher prevalence of the mutation in classical PTC, rather than the follicular variant, and a significantly lower mRNA expression of TPO (*p* <0.0001) in PTCs with BRAF^V600E^ mutation as compared to negative cases, suggesting that BRAF^V600E^-mutated PTCs, although still well differentiated, are losing the typical features of follicular cells. Therefore, this mutation may be related to an early dedifferentiation process rather than advanced PTC stages. In this study we did not analyze TPO expression according to this mutation; however, we are looking forward to starting a new study in this respect.

TPO expression according to TNM stage shows that TPO substantially decreases when TNM increases. The comparison of mean TPO, *Ki67* and *p53* values was statistically significant (*p* <0.01), which corroborates that the immunohistochemical study of TPO has prognostic value, as well as that of *Ki67* and *p53*. However, our results show that an initial analysis of TPO in cytology samples is not effective to distinguish benign from malignant lesions because of TPO expression variability in follicular tumors, consistent with other reports [23, 24]. Nevertheless, TPO may be useful, together with other markers, in confirming or ruling out benign diseases except for low-risk carcinomas such as MIFC.

The relationship between TPO expression and prognosis of thyroid disease has been determined in different studies. Weber, in his 1-year follow-up, observed that 100 % of patients with positive TPO and galectine-3 immunostaining were free of disease at the end of follow-up, compared to 57 % of patients with a negative TPO staining. It was suggested that the continued expression of TPO in cancer might predict clinical outcome, rather than the lack thereof [18]. Pulcrano et al. observed that detectable TPO expression was associated with a lower risk of metastasis in a 5-year follow-up, suggesting that persistent functional differentiation reflects a less aggressive behavior [25]. In the present study, overall survival according to TPO expression showed that patients with negative TPO had decreased survival rates, with a cumulative overall survival of 20 years, but the comparison between groups was not statistically significant (*p* = 0.34). In contrast, survival according to TNM stage was statistically significant (*p* <0.001). Association between disease-free survival and TPO expression was not significant between groups; however, patients with negative or weak TPO levels had a survival of 72 % and 86 %, respectively, in contrast to the 20-year survival rate of 100 % seen with intense TPO expression.

Within the limitations of our study we must underscore the indolent nature of differentiated thyroid cancer and its low mortality rate. Therefore, assessing disease-free survival is challenging since relapses are usually asymptomatic. Nevertheless, this study has detected significant differences in overall survival according to TNM stage in association with TPO levels, and we believe this is due to our sample size and follow-up length, which reached a maximum of 20 years. Our results should be interpreted cautiously since the study may be influenced by a selection bias, as not all patients in the period referred were included in the study. Lastly, the number of thyroid malignancy samples analyzed is limited, especially when considering the subdivisions included in the classification of thyroid tumors, which may be considered an additional limitation.

## Conclusions

In our study, TPO levels represent a useful prognostic factor for differentiated thyroid cancer. It may be highly useful for patient follow-up. A pronounced TPO fall requires assessment because of a high risk for local and/or distant relapse and decreased survival. The difference in TPO expression between MIFC and WIFC is an interesting finding in this study, and should be considered. In view of our results, TPO cannot be considered an effective diagnostic marker to separate benign from malignant disease following fine-needle aspiration in cases of follicular proliferation [18, 19]. Further prospective studies are needed.

## Abbreviations

TPO: Thyroperoxidase
DTC: Differentiated thyroid carcinoma
MIFC: Minimally invasive follicular carcinoma
WIFC: Widely invasive follicular carcinoma
mRNA: Messenger ribonucleic acid
PTC: Papillary thyroid carcinoma
FTC: Follicular thyroid carcinoma
